# Acute Effects of High-Velocity Interval Cycling Versus Continuous Moderate-Intensity Cycling on Cognitive Function in Patients with Mild Cognitive Impairment

**DOI:** 10.3390/brainsci16030342

**Published:** 2026-03-22

**Authors:** Mari Bardopoulou, Costas Chryssanthopoulos, Evgenia D. Cherouveim, Evangelia Tzeravini, Evangelia Stanitsa, Maria Koustimpi, Eirini Chatzinikita, Irini Patsaki, Stelios Poulos, John Papatriantafyllou, Theodoros Vassilakopoulos, Maria Maridaki, Christos Consoulas, Sokratis G. Papageorgiou, Michael Koutsilieris, Anastassios Philippou

**Affiliations:** 1 Medical School, National and Kapodistrian University of Athens, 11527 Athens, Greece; maribardop@med.uoa.gr (M.B.); chryssan@phed.uoa.gr (C.C.); echatzin@med.uoa.gr (E.C.); steliospoulos@gmail.com (S.P.); tvassil@med.uoa.gr (T.V.); cconsoul@med.uoa.gr (C.C.); mkoutsil@med.uoa.gr (M.K.); 2 School of Physical Education and Sports Science, National and Kapodistrian University of Athens, 17237 Athens, Greece; echerouv@phed.uoa.gr (E.D.C.); mmarida@phed.uoa.gr (M.M.); 3 1st Department of Propaedeutic Medicine, Medical School, Laiko General Hospital, National and Kapodistrian University of Athens, 11527 Athens, Greece; evatz90@gmail.com; 4 1st Department of Neurology, “AIGINITEIO” University Hospital, National and Kapodistrian University of Athens, 11528 Athens, Greece; eva.st.92@gmail.com (E.S.); jpapatriantafyllou@gmail.com (J.P.); sokpapa@med.uoa.gr (S.G.P.); 5 Memory Disorders Clinic, Medical Center of Athens Hospital, 15125 Athens, Greece; mariakoustimpi@gmail.com; 6 Physiotherapy Department, Faculty of Health and Caring Sciences, University of West Attica, 12243 Athens, Greece; ipatsaki@uniwa.gr; 7 Third Age Center, “IASIS”, 16562 Athens, Greece

**Keywords:** cognitive function, exercise, high-velocity cycling, mild cognitive impairment

## Abstract

**Highlights:**

**What are the main findings?**

- A single session of high-velocity, low-resistance interval cycling acutely improved global cognition, executive functions, and semantic fluency in patients with mild cognitive impairment (MCI).
- Both high-velocity interval cycling and continuous aerobic cycling elicited cognitive benefits, whereas effects on processing speed and psychomotor vigilance were limited.

**What are the implications of the main findings?**

- Acute aerobic exercise may serve as a feasible non-pharmacological strategy to transiently enhance cognitive performance in MCI.
- High-velocity interval cycling induced comparable cognitive benefits with lower cardiovascular load and perceived exertion.

**Abstract:**

**Background/Objectives:** Physical exercise has emerged as a promising non-pharmacological intervention for cognitive dysfunction; however, the most effective mode of exercise remains unclear. This study aimed to investigate the acute effects of two cycling exercise protocols, (a) continuous aerobic/moderate-intensity (CA) and (b) high-velocity/low-resistance (high-cadence) interval (HVI), on cognitive and executive performance in patients with mild cognitive impairment (MCI). **Methods:** Seventeen patients (10 females and 7 males, age: 65.5 ± 8.85 years) diagnosed with MCI or early-stage Alzheimer’s disease (13 MCI and 4 eAD) participated in a random order in three different conditions: CA, HVI, and control/no exercise (CON). Cognitive parameters were assessed acutely before and after the completion of each condition. **Results:** Significant condition × time interactions were observed for both Montreal Cognitive Assessment (MoCA) and Frontal Assessment Battery (FAB) (*p* < 0.01). Higher scores (*p* < 0.01) for MoCA and FAB post-intervention were found compared to baseline in both exercise bouts, whereas no changes occurred in CON. Interestingly, when post-intervention scores were compared between conditions, cognitive performance was improved only in HVΙ compared to CON in MoCA (*p* < 0.01) and FAB (*p* < 0.001), revealing a stronger acute effect of HVI. **Conclusions:** A single bout of high-velocity, low-resistance (high-cadence) interval cycling acutely enhanced global cognition and executive function in individuals with MCI, exerting greater improvement compared to continuous aerobic exercise or control condition. These findings emphasize the potential utilization of HVI as an effective non-pharmacological intervention to acutely enhance cognitive performance in older adults with MCI.

## 1. Introduction

In recent decades, the prevalence of dementia and cognitive impairment has significantly increased, with one-third of individuals over 65 now presenting dementia or mild cognitive impairment (MCI) [1], reflecting both physiological and pathological changes associated with aging. While cognitive decline is an anticipated feature of normal aging, pathological processes frequently exacerbate these effects, accelerating functional deterioration [2].

The aging of the central nervous system (CNS) is characterized by a reduction in the number of neurons [3,4], diminished cerebral perfusion, and accumulation of metabolic by-products within brain cells [5]. These alterations lead to a progressive decline in functional capacity and cognitive functions, including learning, memory, vision, and hearing, as well as impairments in motor control, coordination, attention, and reaction speed [6]. Notably, the CNS responds more robustly to physical activity than to mental stimulation, maintaining optimal functionality only when its various domains are regularly exercised [7].

MCI occupies a critical position on the continuum between normal aging and dementia, representing a prodromal stage characterized by objectively measurable cognitive deficits that exceed age-related expectations, yet not significantly compromising daily living activities [2,8]. MCI is clinically categorized into three subtypes: amnestic, multi-domain non-amnestic, and single-domain non-amnestic. Patients typically report cognitive complaints corroborated by informants [9] and demonstrate impairments in one or more cognitive domains disproportionate to their age and educational background, while maintaining preserved functional independence [10,11].

The prevalence of MCI increases with age, with epidemiological and meta-analytic evidence indicating that approximately 15–25% of older adults meet criteria for MCI, while the prevalence rises across advancing age groups [12]. Furthermore, individuals with MCI have a substantially higher risk of progressing to dementia compared with cognitively healthy peers, with longitudinal studies reporting annual conversion rates of approximately 10–15%, particularly among adults aged over 65 years [13]. Currently, no pharmacological treatment has been approved for MCI; however, behavioral interventions, including cognitive training and physical exercise, have demonstrated beneficial effects on cognitive performance and functional outcomes [14,15].

Physical exercise may represent a feasible adjunct non-pharmacological strategy for cognitive dysfunction, although the most effective mode of exercise remains unclear [16]. Exercise has been shown to prevent cognitive decline and enhance quality of life (QoL) for individuals with cognitive impairment [17]. The World Health Organization (WHO) recommends 150 min/week of moderate exercise or 75 min/week of higher-intensity exercise to improve both functional capacity and overall health in individuals across all age groups, including those with chronic conditions [1]. Beyond structured exercise programs, everyday physical activity—including occupational, recreational, and sports-related activities—contributes significantly to cognitive, physical, and psychosocial well-being in older adults.

A growing body of research supports the cognitive benefits of exercise, particularly for individuals with MCI [18,19] and during the early stages of neurodegenerative diseases such as Alzheimer’s disease [20], Parkinson’s disease [21], and vascular cognitive impairment [22]. More specifically, high-intensity exercise protocols have shown promise in improving motor and cognitive symptoms in Parkinson’s disease [23,24,25] and cardiovascular function in patients with cardiovascular disease (CVD) and chronic obstructive pulmonary disease (COPD) [26,27]. Nevertheless, studies examining the acute effects of high-intensity or high-speed exercise on cognitive function in patients with MCI or early-stage Alzheimer’s disease are few compared to the studies focusing on the effects of aerobic exercise. Moreover, the immediate effects of a single session of physical exercise on cerebrovascular and cognitive function are not well described so far, and the effects in the healthy older population are largely unknown [5].

To our knowledge, no previous studies have directly compared the acute effects of high-velocity, low-resistance (high-cadence) interval cycling (HVI) and continuous aerobic/moderate intensity (CA) cycling bouts on cognitive and functional performance in individuals with MCI. Therefore, the present study aimed to compare the effects of these two exercise protocols on cognitive and functional outcomes in patients with MCI. We hypothesized that a single session of exercise would elicit acute improvements in cognitive and functional measures in these patients.

## 2. Materials and Methods

### 2.1. Participants

**Figure 1:**
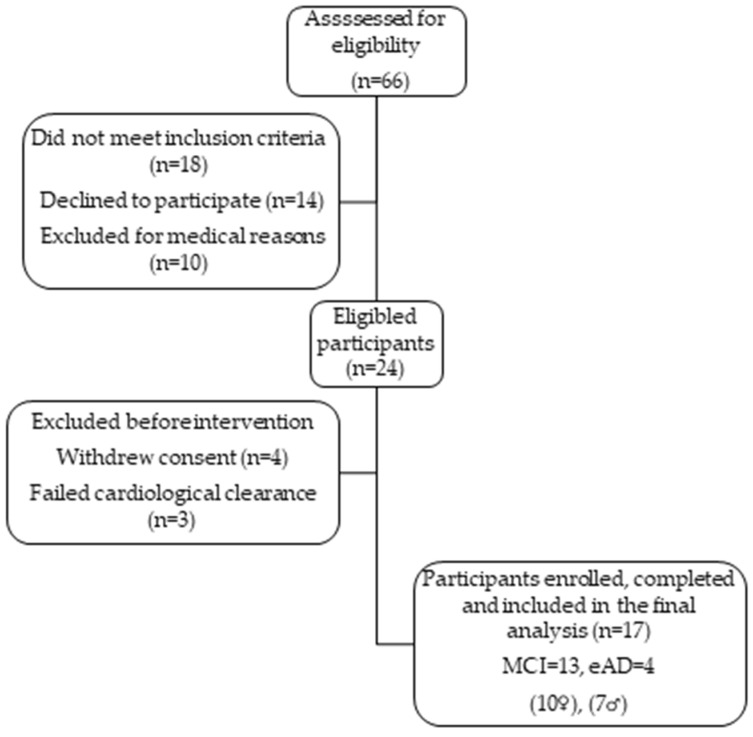
Participant flow diagram illustrating the number of individuals screened, excluded, enrolled, and analyzed throughout the study.

In total, sixty-six outpatients were recruited from the “Memory, Cognitive Disorders, and Rare Dementias” Clinic at the 1st Department of Neurology, “ AIGINITEIO” University Hospital, and referred to the Cardiopulmonary Rehabilitation Program, taking place at the Physiology Laboratory, Medical School. Of these, seventeen participants (13 MCI and 4 eAD) (Table 1) met the following inclusion criteria: (a) diagnosis of MCI according to the criteria in [13] and a Clinical Dementia Rating (CDR) ≤ 0.5 [28], or diagnosis of early-stage Alzheimer’s disease (eAD) with a CDR ≤ 1 [29]; (b) age between 50 and 80 years; (c) functional independence, absence of significant physical limitations, and sufficient communication abilities; (d) medically stable condition for at least six months; (e) cardiological clearance confirming safety for physical activity; (f) and successful completion of cardiopulmonary exercise testing (Cosmed Quark CPET, Rome, Italy) to assess maximal oxygen uptake (VO_2_max). Exclusion criteria included unstable medical conditions, severe mobility limitations, inability to complete CPET, or diagnosis of moderate-to-severe dementia. A flowchart depicting participant screening, eligibility, and enrolment is presented in (Figure 1).

### 2.2. Study Design

Participants performed cycling exercise in two different exercise protocols: high-velocity/low-resistance (high-cadence) interval cycling (HVI), designed to emphasize rapid pedaling under low mechanical load, and continuous aerobic/moderate-intensity cycling (CA). Furthermore, a control condition (non-exercise) (CON) was assigned. The HVI protocol was designed to emphasize maximal pedaling cadence under low mechanical resistance (high-velocity/low-load), prioritizing neuromotor activation rather than high metabolic intensity. Therefore, HVI was expected to elicit lower heart rates and perceived exertion responses compared with CA. The order of the three experimental conditions (HVI, CA, CON) was randomized individually for each participant using a computer random number generator between 1 and 3, where 1 = HVI, 2 = CA and 3 = CON (https://numbergenerator.org, accessed on 15 October 2021). Consequently, HVI was administered first in 6 participants, as the second trial in 4, and as the third trial in 7; CA was conducted first in 9 subjects, as the second trial in 4 and as the third in 4; and CON was performed first in 2 volunteers, second in 9 and third in 6. Experimental sessions (visits 4–6) were completed on separate days, with a 3–7-day interval between sessions, and were scheduled at the same time of day for each participant to minimize circadian-related variability. All participants attended six visits at the Physiology Laboratory of the Medical School of the National and Kapodistrian University of Athens, specifically at the Unit of Molecular and Applied Physiology of Exercise and the Unit of Functional Control of Respiration and Ergospirometry.

During the first visit, participants received a detailed explanation of the study protocol and provided written informed consent. Then, they filled out several baseline tests, such as a questionnaire about their medical history and demographics. To calculate the Body Mass Index (BMI), anthropometric data like stature and body mass were also gathered.

The second visit involved a cardiopulmonary incremental exercise test, which consisted of three continuous 3 min periods of progressive load. After the 3 × 3 min periods, the load was increased every minute until volitional fatigue. The test was performed on an electromagnetically braked cycle ergometer (Ergoline, Ergoselect 4, Bitz, Germany), to evaluate each participant’s VO_2_max and maximum heart rate (HRmax). During the test participants were connected to an open-circuit ergospirometer (Cosmed Quark CPET, Rome, Italy) for the analysis of expired air.

The third visit served as a familiarization visit, during which participants were introduced to the laboratory environment and trained in the use of a cycle ergometer (Monark 874E, Vansbro, Sweden). They also became acquainted with the two exercise protocols to be used during the experimental sessions: high-velocity interval cycling (HVI) and continuous aerobic cycling (CA).

The fourth, fifth, and sixth visits were the experimental sessions, which were carried out on separate days with an interval of 3–7 days between each session. Participants were advised to follow a consistent dietary pattern prior to each visit to minimize possible variability in neurocognitive and functional outcomes. Each session involved one of the three intervention conditions—HVI, CA, or a non-exercise control condition (CON)—assigned in randomized order. All conditions were carried out on the same time of the day.

*High-Velocity Interval Cycling Exercise.*

After a 2 min warm-up at low intensity, each participant performed 10 sets of 10 s sprints at maximal cadence with low resistance (10–20 watts), interspersed with 3 min periods of low-speed cycling (at about 20 revolutions/min) with light resistance (10–15 watts), and concluded with a 2 min cool-down. The total time of the HVI protocol was about 33 min.

*Continuous Aerobic Cycling Exercise.*

After a 3 min warm-up, each participant performed 20 min of continuous cycling at 70% of their HRmax at a pedaling rate of 60 revolutions/min and completed the session with a 3 min cool-down period. The total time of the CA protocol was 26 min.

*Control Condition*

Participants did not engage in physical activity—all participants remained seated in a relaxed state or engaged in casual conversation—for 30 min.

In both exercise conditions, intensity was monitored using heart rate (HR) and the Rating of Perceived Exertion (RPE) scale. Supervision during sessions was conducted by an exercise expert, who was not aware of the purpose of the study.

Participants were instructed to maintain similar sleep and dietary conditions before each visit and to avoid vigorous physical activity the day before and on the day of testing.

### 2.3. Cognitive Assessments

Cognitive performance was evaluated from a clinical neuropsychologist, blind to the experimental treatment, before and after intervention through a comprehensive battery of standardized neuropsychological tests:

*Montreal Cognitive Assessment (MoCA)* was used to assess global cognitive function, including memory, attention, language, visuospatial abilities, executive functions, and orientation [30].

*Frontal Assessment Battery (FAB)* was used to assess executive functions such as conceptualization, mental flexibility, and inhibitory control [31].

*Semantic Verbal Fluency (SVF)*: Participants were asked to produce as many words as possible within 60 s belonging to a specific semantic category (e.g., animals), assessing language fluency and executive function [32].

*Trail Making Test Parts A and B (TMT-A, TMT-B)*: TMT-A measured processing speed and visual attention, while TMT-B assessed cognitive flexibility [33].

*Psychomotor Vigilance Test (PVT)*: A sustained-attention task using the Online Test/Sleep Disorder Center, with a total task duration of 2 min. Participants were instructed to respond as quickly as possible to randomly appearing visual stimuli. Primary outcome measures included mean reaction time (RT), number of lapses (RT > 100 ms), and false responses. Testing was conducted in a quiet room under standardized lighting conditions [34].

The same versions of the neuropsychological tests (MoCA, FAB, and Trail Making Test) were administered across sessions. However, the administration was in a different order each time, before and after every condition.

### 2.4. Functional Tests

Functional performance tests were used to assess participants’ physical abilities, such as muscle strength, mobility, and coordination, in addition to neuropsychological evaluation. Before and after each experimental condition, each functional test was conducted twice, with 60 s of rest in between, and the best score was considered.

***Muscle Strength Tests***

*Upper-limb* strength was evaluated by the Handgrip Strength Test (HG) [35] using a handheld dynamometer (Deyard EH101, electronic hand dynamometer, 90 kg/200 lbs; Shen Zhen Shi Yong Hua E-Commerce Co., Ltd., Shenzhen, China). The participants were seated with their elbow bent at a 90-degree angle and their forearm and wrist in a neutral position. They squeezed the dynamometer as hard as possible, and the device recorded the maximum force exerted.

*Lower-limb* strength and endurance were assessed with the 30-Second Sit-to-Stand Test (30″SST) [36]. Participants were instructed to rise and sit on a chair (seat height: 43 cm) as many times as possible in 30 s, with arms crossed over the chest.

***Mobility Tests***

*Whole-body mobility* was evaluated using the Timed Up and Go (TUG) test [37,38]. The participants seated on a standard chair were instructed to stand up, walk 3 m, turn around, walk back to the chair, and sit down as quickly and safely as possible without using their arms for assistance. The Berg Balance Score (BBS) [39,40] test involved a series of 14 tasks designed to evaluate static and dynamic balance in adults. The Berg Balance Score, ranging from 0 to 56, indicates the level of balance ability and potential fall risk, with lower scores suggesting higher risk.

***Coordination Test***

*Upper-limb* coordination and reaction time were assessed using the Plate Tapping Test (PTT) [41]; the participants were required to tap a designated spot (like a plate or keyboard key) repeatedly, usually with the index finger, and the time for performing 25 consecutive taps was measured.

The primary outcomes of the study were MoCA total and FAB total responses presenting, respectively, global cognition and executive function. All additional cognitive and functional measures were secondary outcomes of the study.

## 3. Statistical Analysis

All data were analyzed using SPSS software (v.25.0; IBM Corp., Armonk, NY, USA). Descriptive statistics (mean ± SE) were calculated for all variables. The normality of the data was confirmed by the Shapiro–Wilk test. A two-way ANOVA with repeated measures on both factors [3 conditions (HVI, CA, CON) × 2 times (pre and post)] was used to evaluate the acute effects of the intervention on cognitive and functional outcomes. Data were also analyzed by order, i.e., Trial 1 vs. Trial 2 vs. Trial 3 × time (pre and post). Furthermore, post–pre condition delta differences were analyzed using one-way ANOVA for repeated measures. Significant main effects and interactions were analyzed using the Bonferroni post hoc test. Effect sizes were calculated using partial eta squared (η^2^) and interpreted as follows: small (η^2^ = 0.01), medium (η^2^ = 0.06), and large (η^2^ ≥ 0.14). The level of statistical significance was set at *p* < 0.05. All assumptions for repeated-measures ANOVA (sphericity, normality) were tested, and Greenhouse–Geisser corrections were applied when necessary.

## 4. Results

### 4.1. Acute Post-Exercise Responses and Cognitive Function (MoCA)

**Figure 2:**
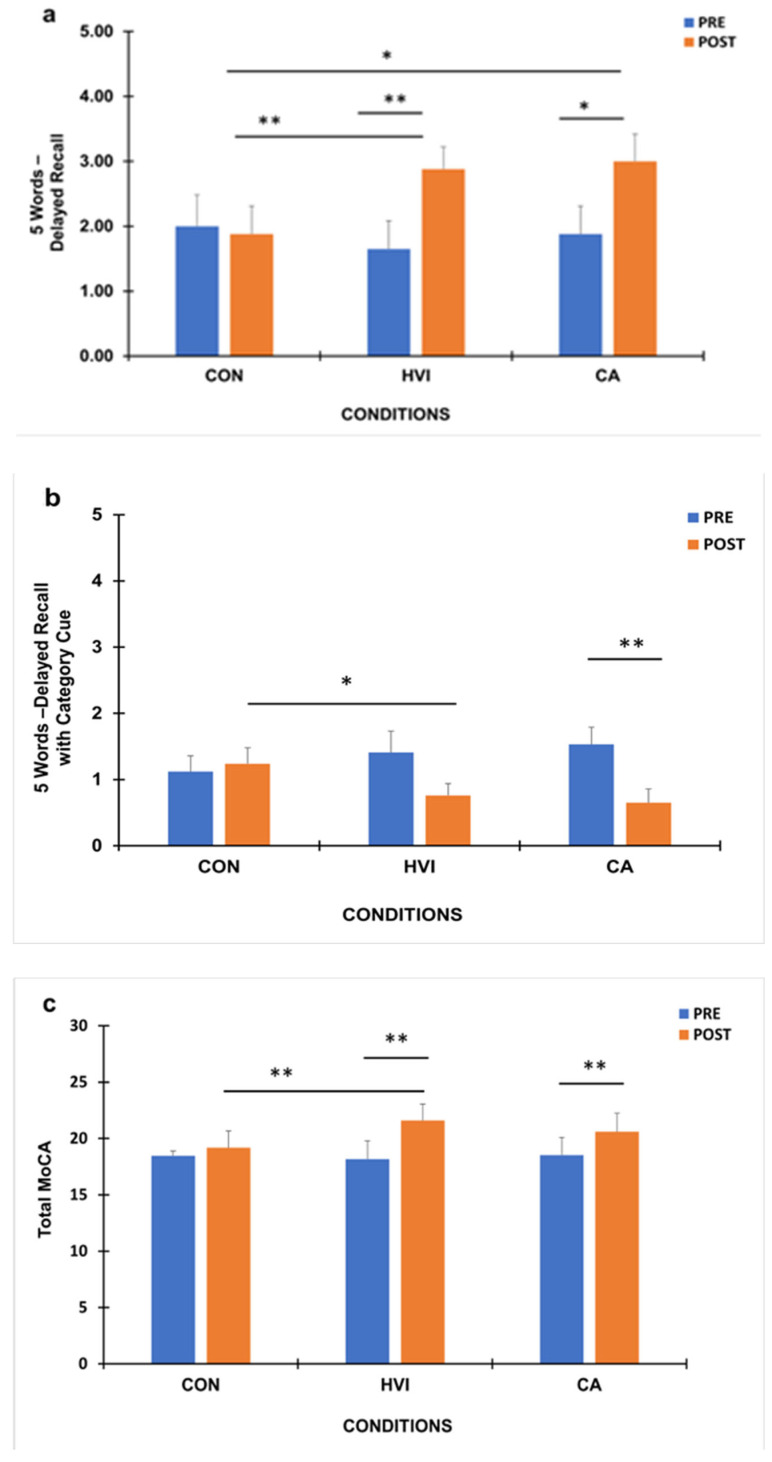
Montreal Cognitive Assessment (MoCA) Delayed Recall (**a**), Delayed Recall Category Cue (**b**) and MoCA total (**c**) scores before (PRE) and after (POST) intervention in the control (CON), high-velocity/low-resistance interval (HVI), and continuous aerobic (CA) conditions (mean ± SE; * *p* < 0.05, ** *p* < 0.01).

Baseline MoCA scores did not differ significantly between conditions (*p* > 0.05) (Figure 2a–c). A two-way repeated-measures ANOVA revealed a significant condition × time interaction for the *5 Words—Delayed Recall* subtest of MoCA, F (2,32) = 6.34 (*p* < 0.01, η^2^ = 0.284) (Figure 2a). Specifically, Delayed Recall scores were improved post-exercise (HVI: 2.88 ± 0.34; CA: 3.0 ± 0.42) compared to pre-exercise levels (HVI: 1.65 ± 0.43; CA: 1.88 ± 0.43) in both HVI (*p* < 0.01) and CA (*p* < 0.05), whereas in CON no change was observed (Pre: 2.0 ± 0.48 vs. Post: 1.88 ± 0.43). Consequently, post-exercise scores were higher in HVI (*p* < 0.01) and CA (*p* < 0.05) compared to CON (Figure 2a). Similarly, delta difference post–pre condition scores were higher in both exercise conditions compared to CON (Table 2).

A significant condition × time interaction was found in the *5 Words—Delayed Recall with Category Cue* subtest of MoCA, F (2,32) = 3.71 (*p* < 0.05, η^2^ = 0.188) (Figure 2b). It should be noted here that lower scores in this category indicate better performance. Post hoc analyses revealed that in the CA condition post-exercise responses were improved compared to pre-exercise (Pre: 1.53 ± 0.26 vs. Post: 0.65 ± 0.21) (*p* < 0.01). In HVI, these scores (Pre: 1.41 ± 0.32 vs. Post: 0.76 ± 0.18) showed a strong tendency for improvement but did not reached significance (*p* = 0.06), whereas in CON no change was recorded (Pre: 1.12 ± 0.24 vs. Post: 1.24 ± 0.24) (*p* > 0.05). On the other hand, comparing post-exercise scores between conditions, only in HVI (*p* < 0.05) was there an improvement compared to CON (Figure 2b). Similarly, delta difference post–pre condition scores were higher only in CA compared to CON (Table 2).

Significant differences were also observed for the most representative outcome in MoCA, that is, the MoCA total score. Specifically, significant condition × time interaction was found for MoCA total score, F (2,32) = 6.59 (*p* < 0.01, η^2^ = 0.292). Post hoc analyses demonstrated that both exercise treatments improved responses post-exercise (HVI: 21.59 ± 1.45; CA: 20.59 ± 1.63) compared to pre-exercise (HVI: 18.18 ± 1.62; CA: 18.53 ± 1.56) (*p* < 0.01), while no change was found in CON (Pre: 18.47 ± 1.57 vs. Post: 19.18 ± 1.48) (Figure 2c). However, when post-exercise scores between conditions were compared, these scores were higher only in HVI compared to CON (*p* < 0.01). Similarly, delta difference post–pre condition scores were higher only in HVI compared to CON (Table 2).

Importantly, no significant pre–post changes were observed in the control condition, arguing against a generalized learning effect.

### 4.2. Acute Post-Exercise Responses and Executive Function (FAB)

**Figure 3:**
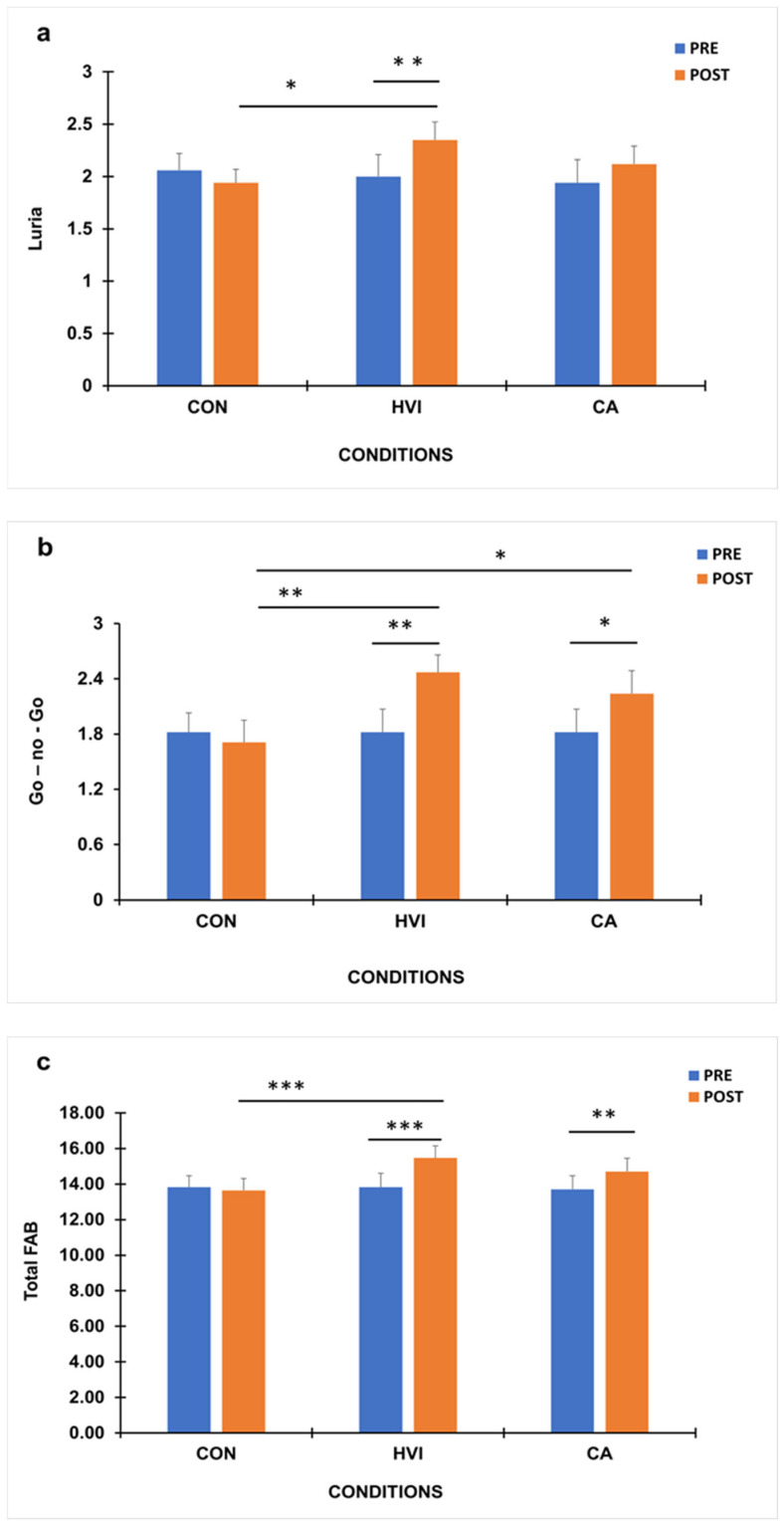
Luria Motor Series (**a**), Go-No-Go (**b**) and Frontal Assessment Battery (FAB) total (**c**) scores before (PRE) and after (POST) intervention in the control (CON), High-velocity/low-resistance interval (HVI), and continuous aerobic (CA) conditions (mean ± SE; * *p* < 0.05, ** *p* < 0.01, *** *p* < 0.001).

Baseline Frontal Assessment Battery scores (FAB) did not differ significantly between conditions (*p* > 0.05) (Figure 3a–c). A significant condition × time interaction was observed for the *Luria Motor Series* subtest of the FAB, F (2,32) = 4.15, *p* < 0.05, η^2^ = 0.21. Post hoc analyses revealed that only the HVI condition produced a significant improvement post-exercise compared to pre-exercise (Pre: 2.00 ± 0.21 vs. Post: 2.35 ± 0.17) (*p* < 0.01), while in the CA (Pre: 1.94 ± 0.22 vs. Post: 2.12 ± 0.17) and CON (Pre: 2.06 ± 0.16 vs. Post: 1.94 ± 0.13) conditions no differences were found (Figure 3a). Consequently, when post-exercise scores were compared only in the HVI condition, these responses were significantly higher (*p* < 0.05) than those in the control group (Figure 3a). Also, when delta difference post–pre condition scores were compared, only HVI was higher compared to CON (Table 2).

A significant main effect of time, F (1,16) = 7.31 (*p* < 0.01, η^2^ = 0.31), and significant condition × time interaction, F (2,32) = 4.97 (*p* < 0.05, η^2^ = 0.24), were found for the *Go–No-Go* subtest of the FAB (Figure 3b). Post hoc analyses revealed that both HVI and CA conditions produced significant post-exercise improvements (HVI: 2.47 ± 0.19; CA: 2.24 ± 0.25) compared with pre-exercise scores (HVI: 1.82 ± 0.25; CA: 1.82 ± 0.25) (*p* < 0.01 and *p* < 0.05, respectively), while the CON condition showed no significant change (Pre: 1.82 ± 0.21 vs. Post: 1.71 ± 0.24) (Figure 3b). Similarly, post-exercise performance in HVI and CA conditions was significantly higher compared to CON (*p* < 0.01 and *p* < 0.05, respectively). However, when delta difference scores were compared, only CA was higher compared to CON despite the fact that this difference was lower than the corresponding value in HVI (Table 2).

Regarding the total score in FAB, significant differences were also recorded (Figure 3c). More specifically, a significant condition × time interaction was found for the FAB total score, F (2,32) = 10.50 (*p* < 0.001, η^2^ = 0.396). Post hoc analyses showed that both HVI and CA conditions led to significant improvements in total FAB performance post-exercise (HVI: 15.47 ± 0.67; CA: 14.71 ± 0.75) compared to pre-exercise values (HVI: 13.82 ± 0.79; CA: 13.71 ± 0.76) (*p* < 0.001 and *p* < 0.01, respectively), whereas no change was observed in the CON condition (Pre: 13.82 ± 0.65 vs. Post: 13.65 ± 0.66) (Figure 3c). However, comparing post-exercise scores, only in the HVI condition were responses significantly higher than those in the control (*p* < 0.001). On the other hand, delta difference post–pre condition scores were higher in both exercise conditions compared to CON (Table 2).

### 4.3. Semantic Fluency (Temporal–Frontal Lexical Retrieval)

A significant main effect of time was observed, F (1,16) = 14.80 (*p* = 0.001, η^2^ = 0.481), indicating higher semantic fluency scores following the session compared with baseline. However, no significant main effect of condition emerged, F (2,32) = 0.63 (*p* = 0.537, η^2^ = 0.038). Similarly, the condition × time interaction did not reach significance, F (2,32) = 2.53 (*p* = 0.096, η^2^ = 0.136). Also, when delta difference post–pre condition scores were analyzed, there were no significant differences in any condition (Table 2).

### 4.4. TMT-A and TMT-B (Processing Speed)

For TMT-A, the repeated-measures ANOVA showed a significant main effect of time, F (1,16) = 7.52 (*p* = 0.014, η^2^ = 0.32), indicating faster post-session performance across all conditions. Although a main effect of condition was observed, F (2,32) = 3.52 (*p* = 0.042, η^2^ = 0.18), post hoc comparisons were not statistically significant (*p* > 0.08). Also, no condition × time interaction emerged, F (2,32) = 0.23 (*p* = 0.795, η^2^ = 0.01).

For TMT-B, neither the main effect of time (F (1,16) = 0.54, *p* = 0.472, η^2^ = 0.03) nor the main effect of condition (F (2,32) = 1.59, *p* = 0.220, η^2^ = 0.09) reached significance. The condition × time interaction approached significance, F (2,32) = 3.84, *p* = 0.056, η^2^ = 0.19, reflecting a trend toward differential pre–post changes among conditions without reaching statistical significance. However, when delta difference post–pre condition scores were analyzed in both TMT-A and TMT-B, there were no differences between conditions (Table 2).

### 4.5. Psychomotor Vigilance Test (PVT)

Performance on the PVT remained stable across conditions and time points. Reaction time duration showed no significant main effect of conditions (*p* = 0.779), time (*p* = 0.835), or condition × time interaction (*p* = 0.909). A similar pattern was observed for false responses, with no significant main effect of condition (*p* = 0.316), time (*p* = 0.889), or interaction (*p* = 0.253). Mean response time also showed no significant differences across conditions (*p* = 0.320) or time (*p* = 0.347), nor a condition × time interaction (*p* = 0.235). Likewise, the number of attempts did not differ significantly between conditions (*p* = 0.821) or across time (*p* = 0.648), and no interaction emerged (*p* = 0.795). Overall, acute exercise—whether high-velocity interval or continuous aerobic—did not elicit measurable changes in vigilance or sustained attention as assessed by the PVT. Also, when delta difference post–pre condition scores were analyzed, there were no significant differences in any condition (Table 2).

### 4.6. Order Effect of Cognitive Data

When cognitive data were analyzed by order (i.e., Trial 1 vs. Trial 2 vs. Trial 3), no significant differences were found at condition or interaction level for any variable. Only time-level differences were observed in MoCA 5-Word Delay Recall (*p* < 0.01, η^2^ = 0.64), MoCA 5-Word Cue (*p* = 0.006, η^2^ = 0.39), MoCA total (*p* < 0.01, η^2^ = 0.80), FAB Go/No-Go (*p* < 0.05, η^2^ = 0.29), FAB total (*p* < 0.01, η^2^ = 0.36), semantic fluency (*p* < 0.01, η^2^ = 0.41) and TMT-A (*p* < 0.01, η^2^ = 0.46).

### 4.7. Functional Performance

**Figure 4:**
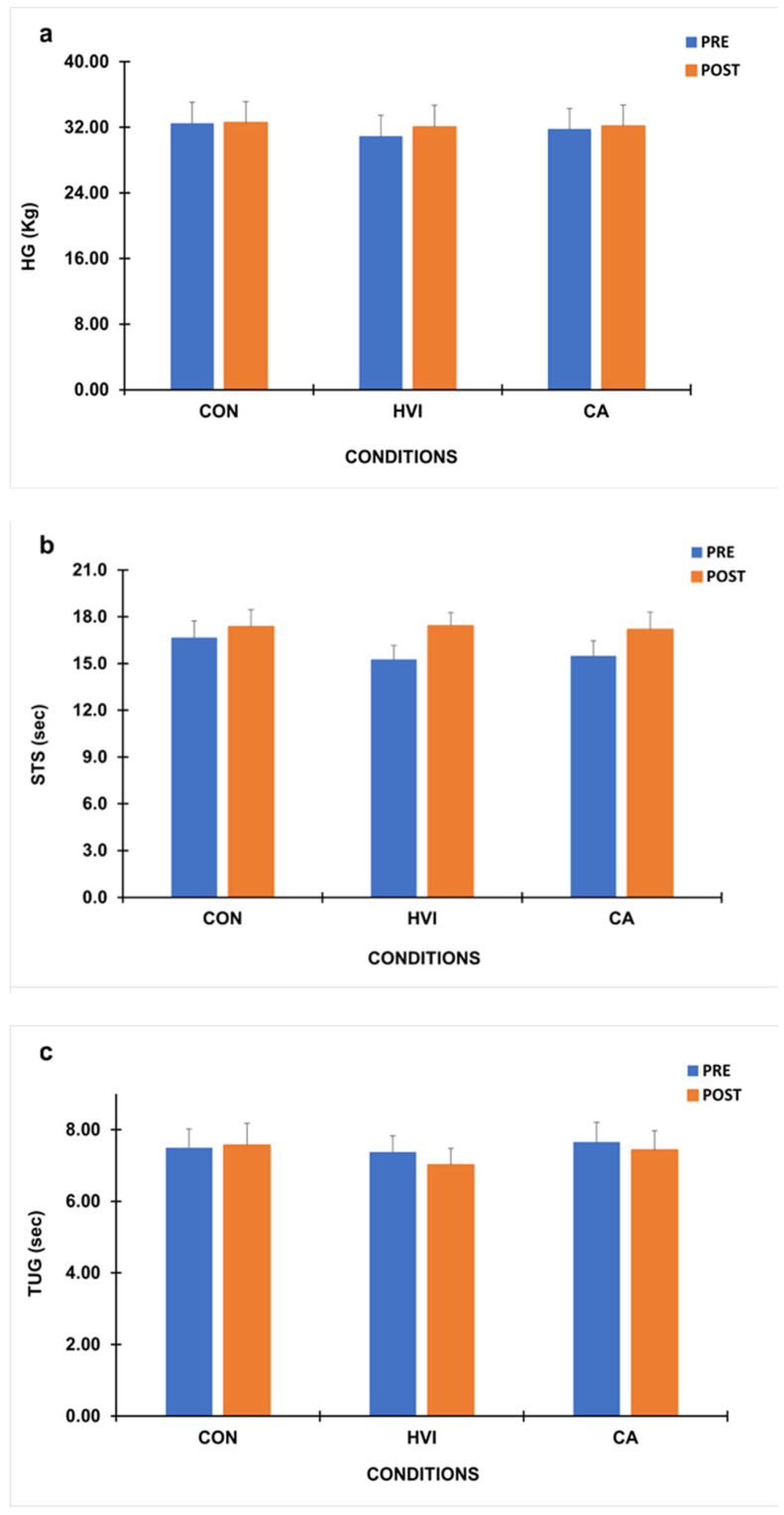

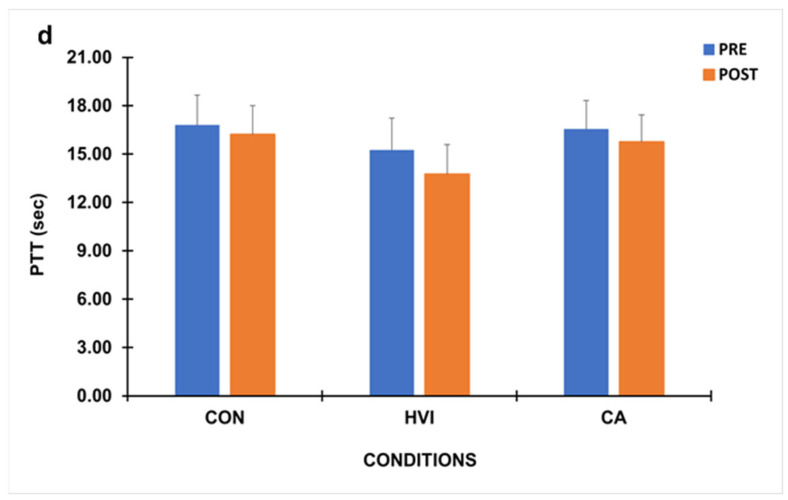
Handgrip (HG) (**a**), 30sec Sit-to-Stand (30s STS) (**b**), Timed Up and Go (TUG) (**c**) and Plate Tapping Test (PTT) (**d**) scores before (PRE) and after (POST) intervention in the control (CON), high-velocity/low-resistance interval (HVI), and continuous aerobic (CA) conditions.

Baseline functional performance did not differ between conditions (*p* > 0.05) (Figure 4). A repeated-measures ANOVA revealed a marginal effect of time for handgrip that did not reach significance, F (1,16) = 4.19 (*p* = 0.057, η^2^ = 0.208), but no significant main effect of condition or condition × time interaction was observed (*p =* 0.073 and *p* = 0.193 respectively). Mean handgrip force values remained stable across all three conditions, with non-statistically significant pre- to post-intervention differences (Figure 4a).

A significant main effect of time was found for the 30sec Sit-to-Stand Test (30s SST), F (1,16) = 15.90 (*p* = 0.001, η^2^ = 0.498) (Figure 4b). Also, no condition effect was found (*p* = 0.65), whereas for condition × time interaction a tendency was observed, F (1.41, 22.52) = 3.91 (*p* = 0.061, η^2^ = 0.18), that did not reach significance (Figure 4b).

No significant effects were observed for Timed Up and Go test (*p* > 0.05). Mean completion times remained stable across all three conditions (CON, HVI, CA), indicating that a single exercise session did not induce measurable changes in mobility or dynamic balance (Figure 4c).

A significant main effect of time was detected for Plate Tapping Test, F (1,16) = 5.21 (*p* = 0.036, η^2^ = 0.246), but neither condition nor the interaction main effects reached significance (*p* > 0.11) (Figure 4d).

Berg balance performance maintained stable before (range: 55.1–55.7) and after intervention (55.4–55.7) in all conditions and the two-way ANOVA revealed no difference at any level (*p* > 0.15). Also, when delta difference post–pre condition scores were analyzed, there were no significant differences in any variable between conditions (Table 3).

### 4.8. Order Effect of Functional Data

When functional data were analyzed by order (i.e., Trial 1 vs. Trial 2 vs. Trial 3) no significant differences were found at condition or interaction level for any variable. Only time-level differences were observed in 30s STS (*p* < 0.01, η^2^ = 0.50) and PTT (*p* = 0.036, η^2^ = 0.24).

### 4.9. Heart Rate (HR) and Rate of Perceived Exertion (RPE) Responses

During the VO_2_max protocol volunteers reached a HRmax of 155 ± 2 b/min and an average VO_2_max of 18.4 ± 1.3 mL/kg/min.

During exercise regarding HR, the two exercise protocols elicited distinct physiological loads. The HVI session produced a mean heart rate of 101 ± 3 b/min, whereas the CA condition resulted in substantially higher values (119 ± 6 b/min) (*p* = 0.001). In HVI average HR during sprints was 111 ± 1 b/min, while during the 3 min low-cadence periods it was 90 ± 2 b/min. Peak cadence in HVI during the 10 s sprints was 101 ± 8 revolutions/min, whereas during the 3 min low-cadence periods it was 21 ± 2 revolutions/min. Peak power output in HVI during sprints was 119 ± 1 watt and during the low-cadence period it was 21 ± 1 watt. On the other hand, in CA pedal speed was constant at 60 revolutions/min, while power output was maintained at 62 ± 7 watt.

RPE followed a similar pattern. Participants reported lower exertion during the HVI session (11 ± 0.3) relative to the CA session (13 ± 0.5) (*p* = 0.05). However, both exercise modalities were well tolerated, even though the CA session imposed a higher acute physiological and perceptual load compared with HVI.

## 5. Discussion

The present study investigated the acute effects of two different cycling protocols, i.e., high-velocity interval cycling (HVI) and continuous aerobic cycling (CA), on cognitive and functional outcomes in individuals with MCI.

### 5.1. Global Cognition and Executive Function

The main findings demonstrated significant improvements from pre- to post-exercise across global cognition (MoCA Delayed Recall and MoCA total) and executive function (FAB total and selected subtests). Significant condition × time interactions indicated that improvements tended to be greater in the exercise conditions (HVI and CA) compared to non-exercise control. Furthermore, HVI induced a higher improvement post-exercise compared to CA, since the most representative cognitive and executive outcomes of total MoCA and FAB responses after exercise were higher only in HVI and not in CA compared to CON. Unlike traditional high-intensity interval training protocols that emphasize metabolic stress, the present HVI intervention was designed to emphasize rapid motor performance under low resistance. Such high-frequency stimuli may temporarily enhance cerebral cortex responsiveness, motor cortex activation, and frontal network involvement through increased sensorimotor drive and afferent feedback. Thus, the cognitive benefits observed after HVI may reflect mechanisms of neuromotor activation rather than pure cardiovascular intensity.

The present findings demonstrated acute improvements in global cognition and memory recall following exercise, as assessed by the MoCA. These results are in line with emerging evidence that a single exercise session can transiently enhance cognitive performance in older adults. Olivio and colleagues [5] reported immediate improvements in cognition and cerebral blood flow after an acute bout of exercise in healthy older adults, while others [25] have found that both high-intensity interval and moderate continuous training acutely elevated BDNF and irisin levels, leading to improved neurocognitive performance in middle-aged and older participants.

Aerobic exercise was found to improve cognitive results in MCI populations in a randomized pilot trial [42], and comparable benefits on cognition and physical conditioning have been reported [18]. More recently, systematic reviews and meta-analyses [8,16] have highlighted aerobic and multimodal exercise as promising strategies for cognitive enhancement in MCI and dementia. Despite the limited number of acute intervention studies in this population, the current findings add to the growing evidence, suggesting that even a single session of exercise may induce measurable cognitive benefits.

In addition to global cognition, the present study demonstrated acute improvements in executive functioning as measured by FAB. Significant condition × time interactions were evident for the Luria Motor Series and Go/No-Go subtests, with greater post–pre improvements following the exercise sessions, especially after HVI, compared with control, while FAB total scores also improved acutely after exercise. These findings suggest that acute exercise may enhance processes related to motor programming, inhibitory control, and prefrontal functioning. Previous work supports this observation [23], reporting improved motor performance and enhanced corticomotor excitability following exercise training in Parkinson’s disease, while high-cadence or high-speed cycling protocols have also been shown to elicit immediate benefits on motor and executive symptoms [21,24]. Within this context, the present findings are compatible with the notion that acute exercise acts as a short-term neuromodulator “boost” to frontal networks, possibly through combined effects on cerebral perfusion, arousal, and neurochemical signaling, as suggested by earlier work on the FAB and frontal dysfunction [32].

Semantic fluency tasks showed a robust main effect of time, with higher post-exercise performance across exercise conditions and descriptive but not statistical larger improvements following HVI. Semantic fluency relies on temporo-frontal networks responsible for lexical access, semantic memory organization, strategic retrieval, and cognitive switching [43], making it a sensitive marker of early neurodegeneration.

The presence of an improvement across sessions suggests contributions from generalized arousal, increased neural efficiency, and potentially enhanced top-down retrieval strategies following exercise. Although the condition × time interaction did not reach significance in semantic fluency, the descriptive superiority of HVI aligns with mechanistic accounts proposing that short bursts of high-velocity movement may induce more potent transient increases in cortical excitability and frontal engagement [22,25]. Importantly, improvements in semantic fluency after a single session have practical relevance, as diminished fluency is not only a diagnostic criterion for MCI but also a strong predictor of progression to Alzheimer’s disease [44,45].

### 5.2. Processing Speed and Vigilance

Processing speed showed a differentiated pattern of responsiveness. For TMT-A, there was a significant main effect of time, indicating faster post-session completion across all conditions, and a main effect of condition without significant post hoc differences, but no condition × time interaction. This suggests a generalized improvement in simple processing speed and visual search that cannot be unequivocally attributed to exercise modality and may partly reflect practice effects or non-specific arousal [46]. The potential influence of learning effects should be recognized, particularly given the short retest interval (3–7 days). A generalized time effect was observed for TMT-A, which may partly reflect learning or non-specific activation. However, this pattern was not consistent across cognitive domains. For MoCA and FAB outcomes, significant condition × time interactions were detected in HVI and CA, whereas no improvement occurred in the control condition or when data were analyzed by order. Thus, although learning effects cannot be fully excluded, the primary outcomes are unlikely to be solely attributable to repeated testing. For TMT-B, which places higher demands on set-shifting and cognitive flexibility, neither the main effects of time nor condition were significant, while the condition × time interaction showed only a non-robust trend. Descriptively, performance tended to worsen in the control session and to improve modestly after both exercise conditions, but this pattern did not reach statistical significance. These findings imply that, in this MCI sample, acute exercise effects on more complex speeded executive tasks are either weaker, more variable, or require larger samples to be reliably detected [47]. This also highlights that not all measures of processing speed are equally sensitive to acute interventions; simpler tasks such as TMT-A may capture generalized activation, whereas TMT-B might require repeated training or higher cognitive reserve to exhibit consistent acute gains [48,49].

In contrast to the cognitive domains that demonstrated measurable acute exercise responsiveness, performance on the PVT remained stable across all conditions and time points. Reaction time, average response time, false responses, and number of attempts showed no significant main effects or interactions, indicating that sustained attention and vigilance were not appreciably influenced by a single bout of exercise in this MCI sample. These findings are consistent with evidence that PVT performance is often characterized by floor effects in error rates and limited intra-individual variability in older adults, reducing its sensitivity to subtle cognitive fluctuations [50,51]. Vigilance tasks rely heavily on basic alertness and simple reaction speed—functions that may require higher arousal thresholds or longer task durations to demonstrate modulation [52]. Meta-analytic findings further suggest that acute exercise predominantly influences executive and memory-related domains rather than low-level vigilance [47,53]. Together, these observations imply that psychomotor vigilance may be less responsive to acute physiological perturbations in MCI patients, particularly when baseline performance is already near optimal and variability is constrained.

Similar neuropsychological batteries have been used in recent exercise intervention studies in older adults and individuals with mild cognitive impairment, including assessments of global cognition, executive function, and processing speed using tools such as the MoCA, Trail Making Test, and verbal fluency tasks [54,55].

### 5.3. Functional Performance

Functional performance outcomes revealed a selective pattern of change. Consistent with previous work, no significant acute effects were observed in handgrip strength or TUG, whereas participants improved in lower-limb strength, as indexed by the 30 s STS, and in manual dexterity, as assessed by the PTT. Handgrip strength has generally shown limited responsiveness to short-term or single-session interventions [35,56], reflecting its dependence on maximal force generation rather than neuromuscular activation patterns that can be rapidly modulated. In contrast, STS is more sensitive to dynamic lower-limb function, balance, and task-specific neuromuscular coordination, which may benefit acutely from increased arousal, muscle temperature, and circulation [36,39,56]. Similarly, manual dexterity tasks such as plate tapping integrate fine motor control with attention and psychomotor speed and may be transiently enhanced by exercise-induced increases in cortical excitability and cerebral blood flow [40]. The absence of significant changes in TUG is consistent with reports that mobility and gait-related outcomes often require longer training periods to show reliable improvements [37,56]. Given the number of secondary and exploratory endpoints examined, findings beyond the predefined primary outcomes should be interpreted with caution.

### 5.4. Physiological Considerations

Physiological responses further contextualize the cognitive and functional findings. As expected, heart rate and perceived exertion were significantly higher during the continuous aerobic session than during HVI, indicating that the continuous protocol imposed a greater sustained cardiovascular and subjective load. Importantly, both protocols were well tolerated, with RPE values falling in the light-to-moderate range for HVI and moderate for CA, suggesting that individuals with MCI can safely complete these exercise bouts. Notably, the more demanding continuous session did not produce clearly superior cognitive or functional benefits compared with HVI, despite its higher heart rate and RPE. This dissociation supports the view that higher physiological strain does not necessarily translate into additional cognitive gains. The present data therefore argue for a pragmatic approach in MCI, prioritizing tolerable, enjoyable protocols that reliably activate, but do not overtax, cardiovascular, metabolic and neurocognitive systems.

### 5.5. Limitations

In the present study several limitations must be acknowledged. The modest sample size (n = 17) is a primary limitation of this study, reducing statistical power for detecting subtle between-condition differences, particularly in interaction effects and in tasks with greater inherent variability. The two exercise protocols were not perfectly matched for total duration and differed primarily in intensity distribution and neuromotor characteristics. Only acute responses to single exercise sessions were examined; thus, the findings cannot be generalized to long-term adaptations or to clinically meaningful trajectories of cognitive decline and functional independence. The absence of neurobiological markers (e.g., BDNF, cerebral blood flow, functional neuroimaging) precludes direct inferences regarding underlying mechanisms. In addition, the presence of potential practice effects, especially in speeded and vigilance tasks, cannot be fully ruled out despite the crossover design and the absence of an order effect. Furthermore, although the administration order of the cognitive tests was different, the fact that no alternate versions were used may have contributed to learning effects in some outcomes as observed at time level in order analysis in some variables such as MoCA, FAB, semantic fluency and TMT-A. Lastly, the study was conducted under controlled laboratory conditions, which may differ from real-world exercise settings in terms of motivation, multitasking, and environmental complexity.

## 6. Conclusions

This study demonstrated that a single session of aerobic exercise—whether performed as HVI or as CA form—can acutely enhance global cognition, specific executive functions, semantic fluency, and selected aspects of functional performance in individuals with MCI. Although the benefits tended to be greater following both exercise bouts compared with control condition, HVI induced a stronger acute response as indicated by total MoCA and FAB data. These findings provide preliminary support for structured low-resistance high-velocity intermittent cycling exercise in the care of individuals with MCI, as a feasible and potentially beneficial adjunct to standard care. Larger trials are required before clinical recommendations can be established. Future studies should employ larger samples, ensure strict time-matching, integrate neurobiological measures, and examine both acute and chronic responses to different exercise modalities and intensities to clarify mechanisms, optimize prescriptions, and determine the sustainability and clinical impact of the observed effects. To our knowledge, this is the first study directly comparing acute high-velocity, low-resistance (high-cadence) interval cycling with continuous aerobic cycling in individuals with MCI, providing novel insight into modality-specific cognitive responsiveness in this population.

## Figures and Tables

**Table 1:** Somatometric, demographic and clinical characteristics of participants (mean ± SE).

Variable	Mean ± SE/n (%)
Number of participants	17
Age (years)	65.5 ± 2.15
Sex (female/male)	10 (59%)/7 (41%)
Body mass (kg)	74.5 ± 3.42
Height (cm)	168 ± 0.02
BMI (kg/m^2^)	26.4 ± 1.14
Diagnosis	MCI: n = 13/eAD: n = 4
CDR score	MCI ≤ 0.5/eAD ≤ 1
Education (years)	11.7 ± 0.8

BMI: Body Mass Index; CDR: Clinical Dementia Rating; MCI: mild cognitive impairment; eAD: early Alzheimer’s disease.

**Table 2:** Post–pre differences in cognitive data in the 3 conditions (mean ± SE).

Variables	CON	HVI	CA
**MoCA 5-word Delayed Recall**	−0.12 ± 0.22	1.24 ± 0.29 *	1.12 ± 0.30 **
**MoCA 5-word Delayed Recall Category Cue**	0.12 ± 0.19	−0.65 ± 0.32	−0.88 ± 0.28 *
**MoCA Total**	0.71 ± 0.51	3.41 ± 0.51 *	2.06 ± 0.52
**FAB Luria Motor**	−0.12 ± 0.08	0.35 ± 0.12 *	0.18 ± 0.15
**FAB Go-No-Go**	−0.12 ± 0.19	0.65 ± 0.19	0.41 ± 0.17 *
**FAB Total**	−0.18 ± 0.23	1.65 ± 0.38 **	1.0 ± 0.33 *
**Semantic Fluency**	0.00 ± 0.61	2.12 ± 0.48	0.82 ± 0.70
**TMT-A**	−24.42 ± 16.88	−34.38 ± 12.28	−27.13 ± 11.04
**TMT-B**	22.41 ± 15.93	−18.59 ± 6.63	−14.68 ± 6.47
**PVT Reaction Time**	0.12 ± 0.56	0 ± 0.62	−0.29 ± 0.68
**PVT False Response**	0 ± 0.15	−0.06 ± 0.16	0.12 ± 0.15
**PVT Mean Response**	−11.76 ± 55.84	83.94 ± 44.28	30.35 ± 41.18
**PVT Number of Attempts**	0.59 ± 1.50	0.12 ± 1.19	−0.59 ± 0.54

* *p* < 0.05 from CON; ** *p* < 0.01 from CON.

**Table 3:** Post–pre differences in functional data in the 3 conditions (mean ± SE).

Variables	CON	HVI	CA
**HG**	0.08 ± 0.57	1.16 ± 0.42	0.40 ± 0.27
**30s STS**	0.7 ± 0.7	2.2 ± 0.3	1.7 ± 0.4
**TUG**	0.09 ± 0.17	−0.35 ± 0.19	−0.22 ± 0.13
**PTT**	−0.58 ± 0.55	−1.48 ± 0.46	−0.78 ± 0.44
**BBS**	0.1 ± 0.1	0.3 ± 0.2	0.0 ± 0.2

BBS = Berg Balance Score.; PTT = Plate Tapping Test; STS = Sit-to-Stand; TUG = Time Up and Go.

## Data Availability

The original contributions presented in this study are included in the article. Further inquiries can be directed to the corresponding author.
